# Acetic Acid as Processing Aid Dramatically Improves Organic Solvent Solubility of Weakly Basic Drugs for Spray Dried Dispersion Manufacture

**DOI:** 10.3390/pharmaceutics14030555

**Published:** 2022-03-02

**Authors:** Molly S. Adam, Warren K. Miller, Amanda M. Pluntze, Aaron M. Stewart, Jonathan L. Cape, Michael E. Grass, Michael M. Morgen

**Affiliations:** Global Research & Development, Lonza, Bend, OR 97703, USA; molly.adam@lonza.com (M.S.A.); warren.miller@lonza.com (W.K.M.); amanda.pluntze@lonza.com (A.M.P.); aaron.stewart@sagerx.com (A.M.S.); jon.cape@lonza.com (J.L.C.); michael.grass@lonza.com (M.E.G.)

**Keywords:** spray drying, pharmaceuticals, poor solubility, processing aid, manufacturability, acetic acid, brick dust compounds, amorphous solid dispersion, sustainable, ionized drug

## Abstract

Many active pharmaceutical ingredients (APIs) in the pharmaceutical pipeline require bioavailability enhancing formulations due to very low aqueous solubility. Although spray dried dispersions (SDDs) have demonstrated broad utility in enhancing the bioavailability of such APIs by trapping them in a high-energy amorphous form, many new chemical entities (NCEs) are poorly soluble not just in water, but in preferred organic spray drying solvents, e.g., methanol (MeOH) and acetone. Spraying poorly solvent soluble APIs from dilute solutions leads to low process throughput and small particles that challenge downstream processing. For APIs with basic p*K*_a_ values, spray solvent solubility can be dramatically increased by using an acid to ionize the API. Specifically, we show that acetic acid can increase API solubility in MeOH:H_2_O by 10-fold for a weakly basic drug, gefitinib (GEF, p*K*_a_ 7.2), by ionizing GEF to form the transient acetate salt. The acetic acid is removed during drying, resulting in a SDD of the original GEF free base having performance similar to SDDs sprayed from solvents without acetic acid. The increase in solvent solubility enables large scale manufacturing for these challenging APIs by significantly increasing the throughput and reducing the amount of solvent required.

## 1. Introduction

It has been widely reported that an increasing fraction of new chemical entities (NCEs) in the pharmaceutical pipeline are poorly water soluble, and that many require solubilization technology to achieve high oral bioavailability [1,2]. Among several formulation approaches to improve the oral bioavailability of these challenging compounds, amorphous solid dispersions (ASDs) are particularly enabling due to their ability to substantially increase dissolved drug concentrations, with enhancements ranging from 2 to 100 times the crystalline solubility, which can significantly increase absorption [3,4,5,6,7,8,9]. In recent years, spray drying has become the most common manufacturing technology for commercial production of pharmaceutical ASDs due to its broad applicability, scalability, and low temperature exposure due to evaporative cooling. There are currently at least 20 commercial drug products that utilize spray dried dispersions (SDDs) [10,11,12,13,14].

Spray drying an ASD entails dissolving an active pharmaceutical ingredient (API) and (typically) polymeric excipient(s) in an organic solvent followed by atomization and droplet drying through contact with heated gas. Acetone and methanol (MeOH) are two preferred spray solvents, due to their high volatility, ability to dissolve a wide range of APIs and useful dispersion polymers, relatively low solution viscosity, equipment compatibility, and safety and environmental considerations [15]. The maximum dissolved solids concentration in the spray solution can be limited by the maximum viscosity for atomization, or by the solubility of the drug, or of the dispersion polymer or other formulation excipients in the spray solvent [16]. With an increasing number of NCEs exhibiting poor organic solubility, including many ‘brick dust’ APIs having very low solubility (e.g., <~1 wt%) in most solvents, the dissolved solids concentration in the spray solution is frequently limited by API solubility. Low API concentration in the spray solvent leads to low manufacturing throughput, resulting in high cost, which can render the product uneconomical to produce. Spray drying of dilute solutions leads to very small particles that are nonoptimal for downstream processing and can adversely affect product performance [17,18].

There are several approaches to address poor spray solvent solubility, including use of alternative solvents or heated solvents. Although THF (tetrahydrofuran) and DCM (dichloromethane) are better at solubilizing some APIs, THF is not used at scale for spray drying due to the explosion hazard resulting from potential peroxide formation, and it can also oxidize API in solution. DCM is generally not desirable from an environmental perspective, as chlorinated solvent emission is becoming more heavily regulated [19], and requires additional safety precautions for operators during manufacturing [20]. In addition, compared to some other solvents, DCM results in significantly more viscous solutions, which limits the usable solution concentration range irrespective of API solubility.

Heating a solvent to a temperature below its boiling point, e.g., 40–50 °C for methanol or acetone, can modestly increase the API solubility. For more significant increases in solubility, a suspension of API can be heated to temperatures above the ambient boiling point using an in-line heat exchanger to rapidly increase temperature [21,22]. Although often feasible, this superheated process is more complex than the typical spray drying process and can also cause chemical degradation for some APIs due to the high temperature exposure.

An approach applicable to ionizable APIs is to form a salt, having high solubility in the preferred spray solvents. Salt formation is a common technique to increase drug solubility and dissolution rate in aqueous media [23], and can also be used to increase API solubility in polar protic solvents such as MeOH. It has previously been shown that alkali metal hydroxides can be used to form alkali metal API salts to increase solubility in polar protic solvents to improve SDD manufacturability for acidic APIs [24]. This approach results in isolation of the metallic salt, or at least in a dispersion containing the metal ions. In addition, we have previously shown that lipophilic acids can be used to form salts of basic APIs in organic spray solvents such as acetone [25]. Like the metal ions, these lipophilic acids are not volatile and remain in the SDD after drying. Despite the potential for increasing solvent solubility using metallic or lipophilic salts, the nonvolatile counter ions add extra mass to the dispersion, decreasing drug loading in the tablet. Additionally, the metallic salts can increase SDD hygroscopicity, potentially decreasing physical or chemical stability. For these reasons, it is often preferred to have the free form of the API in the final dosage form. For weakly basic drugs, a volatile acid can be used to impart higher API solubility in the spray solvent through ionization, but allowing the API to convert back to the free form after drying by losing the volatile acid. Acetic acid is well suited for this process because it has a moderate p*K*_a_ (4.75) and thus is not as tightly bound to a weakly basic API compared to a stronger acid such as HCl (p*K*_a_ = −6) which would not be expected to dissociate from the basic API due to strong bonding.

**Figure 1:**
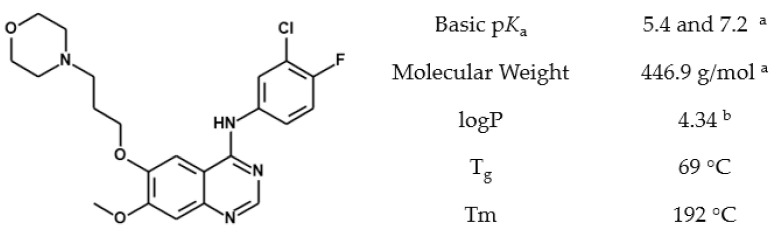
Gefitinib chemical structure and physicochemical properties. ^a^ Reference [26]. ^b^ Calculated using ADMET Predictor™ (Simulations Plus, Lancaster, CA, USA) version 10.

In this study we describe using acetic acid as a processing aid to form the higher solubility ionized form of gefitinib (GEF) (Figure 1), to increase its solubility in MeOH for spray drying. SDDs of GEF with hydroxypropyl methyl cellulose (HPMC) and hydroxypropyl methylcellulose acetate succinate (HPMCAS) were manufactured using MeOH:H_2_O mixtures with acetic acid as a processing aid, and separately using tetrahydrofuran (THF):H_2_O as a control. The resulting SDDs were characterized and compared.

## 2. Materials and Methods

### 2.1. Material Sourcing

GEF (CAS 184475-35-2) was purchased from LC Laboratories (Woburn, MA, USA). Hydroxypropyl methylcellulose acetate succinate (HPMCAS) MG grade was purchased from Shin-Etsu Chemical Co., Ltd. (Tokyo, Japan). Hydroxypropyl methylcellulose (HPMC) E3 Prem grade was purchased from DOW chemical (Midland, MI, USA). Glacial acetic acid was purchased from Fisher Chemical (Hampton, NH, USA) and MeOH, Acetonitrile (ACN) and THF were purchased from Honeywell International, Inc. (Morris Plains, NJ, USA). Hydrochloric acid, sodium phosphate, potassium phosphate, sodium chloride, deuterated MeOH, acetic acid, and water were purchased from Sigma-Aldrich (St. Louis, MO, USA).

### 2.2. Gefitinib Solubility

Crystalline GEF was added in excess (100 mg/mL) to MeOH and MeOH:H_2_O mixtures with varying amounts of acetic acid to form saturated solutions at 20 °C. Unless otherwise stated, all solvent ratios are by mass. After 1 h of stirring, 1 mL aliquots were centrifuged at 10,000 relative centrifugal force (RCF) for 3 min. The supernatant was diluted using 5:1 ACN:H_2_O and analyzed on an Agilent 1290 Infinity II UPLC (Agilent Technologies, Santa Clara, CA, USA) with 0.1% Trifluoroacetic acid (TFA) in 50:50 ACN:H_2_O mobile phase run isocratically at 1 mL/min for 2.5 min per injection with a 5 μL injection volume on an Agilent Zorbax SB-C18 column (4.6 mm × 50 mm with 3.5 μm pore size) set at 35 °C. Analysis was performed at 332 nm with GEF quantified against a standard curve prepared between 4 and 413 µg/mL. Solids were removed from the saturated solutions after 13 days and placed directly onto PXRD cups for analysis of the wet cakes.

### 2.3. pK_a_ Assessment by 1H-NMR

The proton transfer from acetic acid to GEF in MeOH and MeOH:H_2_O mixtures was assessed by proton nuclear magnetic resonance (1H-NMR). First, a calibration curve of chemical shift vs. fraction GEF protonated was made in deuterated solvent. For this, deuterated stock solutions were prepared by combining 1 mL of acetic acid-d4 with 9 mL of either MeOH-d4 or 80:20 MeOH-d4:D2O. GEF (6 mg) was accurately weighed into 4 mL scintillation vials. Solvent (2 mL) was added as a combination of the acetic acid stock solutions and the corresponding MeOH-d4 or 80:20 MeOH-d4:D2O, such that a series of solutions had acetic acid content of 0, 0.5, 1.2, 1.9, 5, 20, and 100 molar equivalents relative to GEF. The GEF concentration in these calibration measurements was below the free-base solubility, resulting in complete dissolution of all components.

The resonance shifts of two GEF peaks, 2.12 and 3.72 ppm, were recorded as a function of acetic acid content. Given rapid proton exchange, these resonances were assumed to track the average chemical shift, which would be expected to vary linearly with the degree of GEF protonation. The data were used to make titration curves to provide estimates for degree of protonation from the solubility experiments described above (Section 2.2).

For all experiments, samples were transferred into 5 mm borosilicate NMR tubes and analyzed by 1H-NMR using a Varian 600 VNMRS spectrometer (Varian Medical Systems UK, Crawley, UK). Eight scans were performed on each sample. Samples in protic solvent were run unlocked with the wet-1D pulse sequence to suppress solvent signals. Spectra were appropriately phased, baseline subtracted, and referenced to the MeOH resonance at 3.31 ppm.

### 2.4. Spray Dry Manufacturing

Table 1 summarizes spray solutions used to prepare 25:75 GEF:polymer SDDs. Spray solutions with acetic acid were prepared by first dissolving the polymer in a mixture of MeOH and water, then adding GEF to form a slurry. Acetic acid was added to this slurry to dissolve the drug.

Control SDDs (lots 1B, 2B) were prepared by first dissolving GEF in THF:H_2_O and then adding polymer. THF-based mixtures were used to process the control SDDs due to the high solubility of GEF in THF. A higher proportion of water was needed for formulation 2B to dissolve the HPMC. The dissolved solids concentration was kept constant between SDDs sprayed from MeOH: H_2_O with acetic acid vs. THF:H_2_O.

Spray drying was performed at laboratory scale on a custom-built dryer with 35 kg/h of nitrogen flowrate capacity. A two-fluid nozzle ¼ J series with a 1650 liquid body and a 64 air cap (Spraying Systems Company, Wheaton, IL, USA) was used to atomize the droplets with an atomization gas pressure of 15–20 psi and a solution flowrate of 15 g/min. The inlet nitrogen flowrate was 450–500 g/min, and the inlet temperature was adjusted to maintain an outlet dryer temperature of 45–50 °C.

### 2.5. Secondary Drying

Secondary drying was performed to remove excess solvent to below ICH limits. Each SDD lot was split into two and a portion dried at each of two conditions (40 °C/15% relative humidity (RH) and 60 °C/30% RH) in a tray dryer (Model ES2000, Bahnson Environmental Specialties, Raleigh, NC, USA). Samples were taken at several time points from 0 to 24 h during drying and analyzed by headspace gas chromatography on an Agilent 7890 GC equipped with a 7697 headspace autosampler. GC parameters are in the Supplementary Material S1.

### 2.6. Powder X-ray Diffraction (PXRD)

To confirm a lack of GEF crystals, XRD was performed with a Rigaku MiniFlex 600 X-ray diffractometer (Rigaku, Tokyo, Japan) equipped with a copper anode (K_α1_ = 1.5406 Å; K_α2_ = 1.5444 Å) generator at 40 kV and 15 mV and a D/teX ultra-high speed detector. Samples were loaded onto 0.2 mm deep Si (510) “zero background” cups and scanned over the 2-theta range 3–40° 2θ, at a rate of 2.5°/min (dispersions) or 10°/min (pure GEF samples) in continuous scanning mode.

### 2.7. Differential Scanning Calorimetry (DSC)

DSC was performed to confirm that the SDDs had a single glass transition temperature (T_g_), indicative of a homogeneous dispersion. Thermograms were collected on a TA instrument, Q2000 DSC (TA instruments, New Castle, DE, USA), from 20 to 160 °C at 2.5 °C/min with a ±1.5 °C/min modulation. The T_g_ was taken as the midpoint of the transition inflection on the reversing heat flow curve. Approximately 5 mg of SDD was placed in a Tzero pan (TA instruments, New Castle, DE, USA) with Tzero lid placed on the sample using a press. Samples were held at 40 °C overnight before analysis to remove any trace solvent. Samples were prepared and analyzed in triplicate.

### 2.8. Scanning Electron Microscopy (SEM)

To assess the particle morphology, the SDD was imaged using a Hitachi SU3500 SEM (Hitachi ltd., Tokyo, Japan) at 15 keV accelerating voltage with secondary electron detection. Samples were adhered to an aluminum specimen stub with a conductive carbon adhesive tab and sputter coated with AuPd for 7 min at 15–20 mAmp plasma current using an Anatech Hummer 6.2 sputter coater (Anatech, Garfield, NJ, USA).

### 2.9. In Vitro Dissolution Testing

In vitro dissolution testing was performed to assess the potential impact of acetic acid as a processing aid on SDD solubilization performance. Pion Rainbow™ fiber-optic UV probe detection (Pion Inc., Billerica, MA, USA) was used to measure GEF concentrations throughout the dissolution test. A simulated gastric to simulated intestinal transfer test was used, in which the pH values of the media were chosen to be physiologically relevant, and the drug concentration was chosen to achieve non-sink conditions of the amorphous GEF in the simulated intestinal media. Prior to collecting dissolution data, unique calibration curves were generated for each UV probe (2-mm path length) by adding a stock GEF solution (32 mg/mL dissolved in DMSO) to 50 mL of 0.01 N HCl (pH 2) and 67 mM phosphate buffer (pH 6.5), respectively. Media (10 mL) was added to the dissolution vessel containing SDD powder to achieve a GEF concentration of 1 mg/mL. Samples were stirred at 100 rpm and held at 37 °C by circulating water through a heating block mounted to a Pion µDiss™ profiler. Dissolution performance in 0.01 N HCl was monitored for 30 min at 386–390 nm using second derivative spectra within a calibration range of 0 to 1084 µg/mL After 30 min, 0.01 N HCl simulated gastric media was diluted 1:1 with 10 mL 133 mM phosphate buffer (i.e., double the final concentration to account for dilution) to a concentration of 500 µg/mL GEF. Dissolution performance in 67 mM phosphate buffer was monitored for 90 min at 300–310 nm using second derivative spectra within a calibration range of 0 to 318 µg/mL. All samples were analyzed in duplicate.

## 3. Results

### 3.1. Solubility

**Figure 2:**
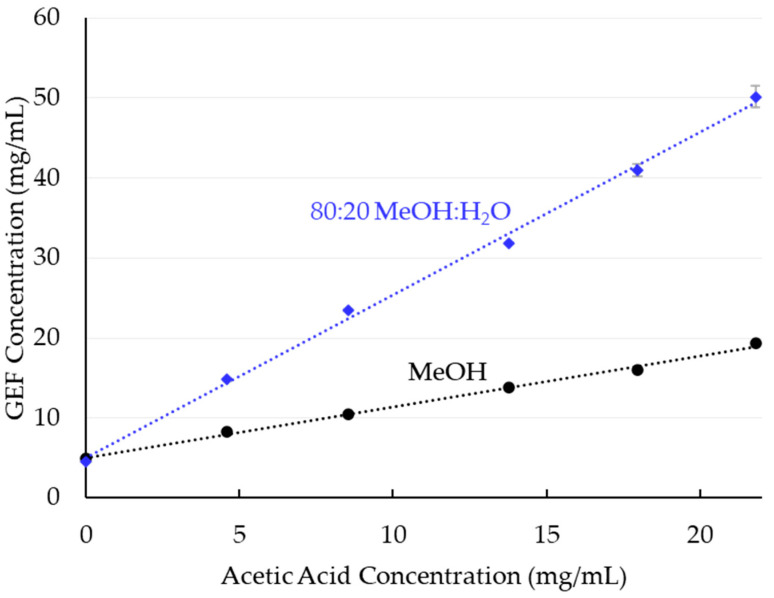
Solubility of GEF in MeOH and 80:20 MeOH:H_2_O as a function of acetic acid concentration.

The solubility of GEF in MeOH and 80:20 MeOH:H_2_O was measured as a function of acetic acid content by adding various amounts of acetic acid to solvent slurries containing excess GEF solids. The solubility increased from 5.0 mg/mL in pure MeOH to 19.3 mg/mL with the addition of 21.8 mg/mL acetic acid (ca. 8 molar equivalents relative to the solubilized GEF). In 80:20 MeOH:H_2_O, GEF solubility increased about 10-fold from 4.6 mg/mL without acetic acid to 50 mg/mL with 21.8 mg/mL acetic acid (ca 3–4 molar equivalents relative to the solubilized GEF) (Figure 2). The remaining solids from most samples are consistent with the methanol hemisolvate (Form 2) of GEF [27] (Figure S1 and Table S1). At the highest acetic acid concentration and in the 80:20 MeOH:H_2_O samples containing acetic acid, additional peaks were observed in the diffractograms of the solids. This may result from precipitation of an additional solid form between sampling and analysis (Figure S2 and Table S2).

### 3.2. Gefitinib Protonation by Acetic Acid

To confirm that the GEF solubility increase with added acetic acid is due primarily to ionization of the drug, the extent of protonation of GEF was determined by NMR. First, titration curves for acetic acid and GEF in deuterated solvent systems were obtained (Figure S3), showing a change in chemical shift with acetic acid due to protonation of GEF, and allowing determination of fraction GEF protonated in solubility experiments. Using the resulting calibration of chemical shift versus fraction GEF protonated, NMR chemical shifts were measured for two points (two acetic acid:GEF ratios) of each curve in Figure 2 to understand the impact of solvent composition on the proton transfer equilibrium between drug and acid. The results are shown in Table 2.

The fraction ionized was used in combination with the total (ionized plus non-ionized) drug solubility to calculate the equilibrium constant (Equation (1)) of the acid-base reaction between GEF and acetic acid in MeOH and 80:20 MeOH:H_2_O:

$${\mathrm{CH}}_{3}\mathrm{OOH}+D\;\to {\;\mathrm{CH}}_{3}{\mathrm{OO}}^{-}+{\mathrm{DH}}^{+}$$

(1) $$K=\frac{\left[{\mathrm{CH}}_{3}{\mathrm{OO}}^{-}\right]\left[{\mathrm{DH}}^{+}\right]}{[{\mathrm{CH}}_{3}\mathrm{OOH}]\left[D\right]},$$

where D and DH^+^ denote the free base and protonated form of GEF, the bracketed entities represent molar concentrations, the acetate concentration is assumed to be equal to the ionized drug concentration, and the equilibrium constant for the proton exchange is *K* = *K*_a_/*K*_d_, where *K*_a_ and *K*_d_ are the acid dissociation constants for acetic acid and the conjugate acid of the drug, respectively. The p*K*_a_ difference (Δ p*K*_a_) between GEF and acetic acid in the solvent is shown in Equation (2):

(2) $$\Delta \;pK_{a}=pK_{a,d}-pK_{a,a}=\log K,$$

where p*K*_a,d_ and p*K*_a,a_ are the p*K*_a_ values for the conjugate acid of GEF and of acetic acid, respectively. The ∆ p*K*_a_ values are shown in Table 2.

The drug solubility enhancement (*Enh*) is the sum of the concentrations of ionized and nonionized dissolved drug divided by the solubility of the unionized drug shown below in Equation (3):

(3) $$\mathrm{Enh}=\frac{\left[{\mathrm{DH}}^{+}\right]+{\left[D\right]}_{s}}{{\left[D\right]}_{s}},$$

where [D]_s_ is the molar free base drug solubility in the spray solvent.

### 3.3. Solvent Removal

Each SDD lot was split, and one portion was dried at 40 °C/15% RH and one at 60 °C/30% RH for 24 h to remove residual solvents. Acetic acid removal was monitored during the course of drying.

**Figure 3:**
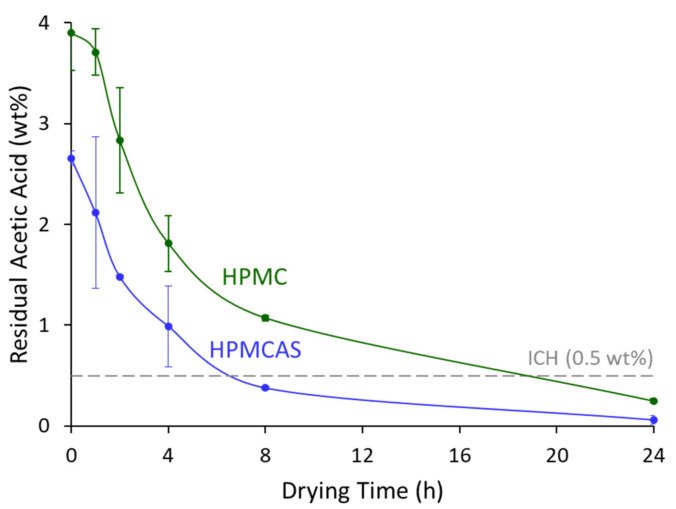
Acetic acid drying curves of the GEF SDDs of HPMC (green) and HPMCAS (blue) dried at 40 °C/15% RH.

At 40 °C/15% RH, acetic acid was removed rapidly to below its ICH limit of 0.5 wt% from both HPMCAS and HPMC-based SDDs, resulting in acetic acid levels of 0.06 and 0.25 wt%, respectively, at 24 h shown in Figure 3 [28]. Residual methanol before secondary drying was 0.70 and 0.78 wt% for the HPMCAS and HPMC SDDs, respectively, and was removed to below the detection limit of 0.01 wt% and well below its ICH limit of 0.3 wt% (3000 ppm) within one hour for both samples [28]. Drying curves for 60 °C/30% RH exhibited more rapid solvent removal as shown in Figures S4 and S5. These more aggressive conditions were not required in the present case due to sufficient drying kinetics at the less aggressive conditions associated with Figure 3. All characterizations on the SDDs presented below were completed with the material secondary dried at 40 °C/15% RH.

Residual THF in the control samples at 24 h drying was 0.045 and 0.004 wt% for HPMCAS and HPMC control SDDs, respectively, which is well below the ICH limit of 0.072 wt% [28].

### 3.4. SDD Characterization

The physical properties and in vitro dissolution performance of SDDs manufactured using acetic acid as a processing aid in MeOH:H_2_O were compared to control SDDs sprayed from THF:H_2_O.

**Figure 4:**
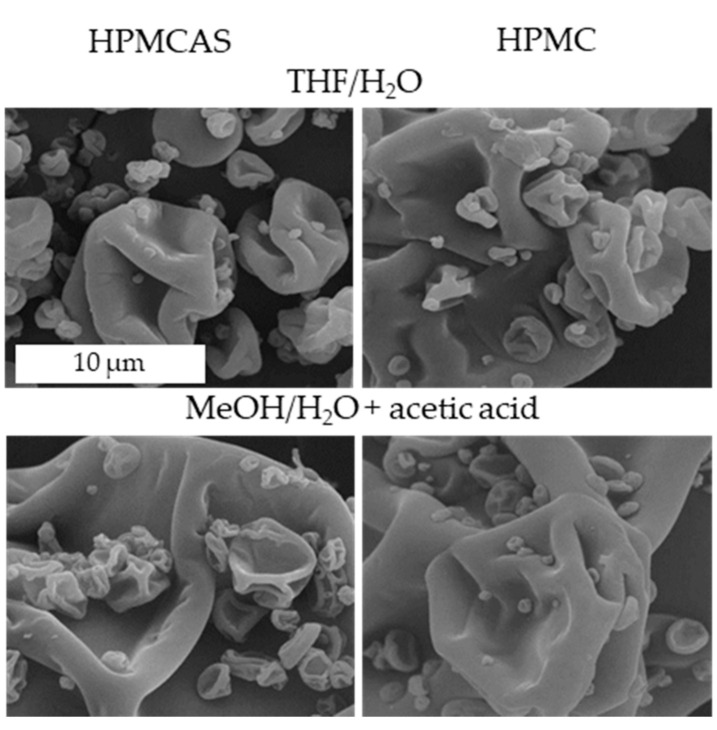
Representative SEM images of the 25% GEF SDDs with HPMCAS (**left**) and HPMC (**right**) standard SDDs (**top**) and acetic acid processing (**bottom**). The scale is the same for all images.

Figure 4 shows that all lots had typical morphology for SDDs of these polymers and drug loadings, consisting mainly of collapsed spheres.

**Figure 5:**
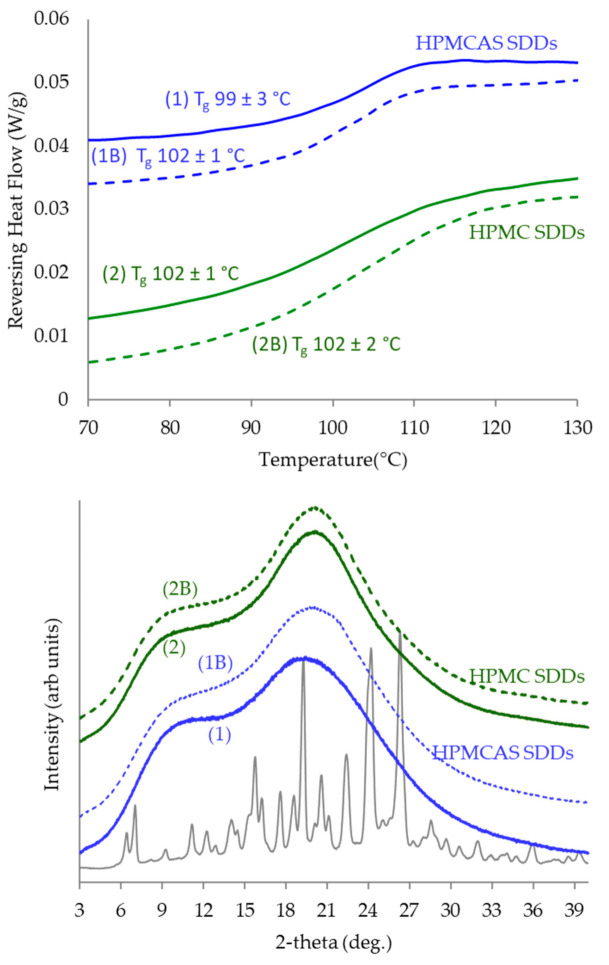
PXRD diffractograms (**bottom**) and DSC reversing heat flow (**top**) of the HPMC (green) and HPMCAS (blue) GEF SDDs sprayed with acetic acid compared to the controls (dashed lines). *Y*-axis is offset for clarity. The crystalline API lot used to manufacture the SDDs is shown for reference in the PXRD plot. DSC non-reversing and total heat flow for SDDs are shown in Figure S8.

All samples resulted in homogeneous amorphous solid dispersions, as evidenced by the lack of surface crystals in the SEM images of Figure 4, the lack of diffraction peaks in the PXRD, and a single T_g_ in the DSC scan, as shown in Figure 5. The dispersion T_g_ lies between that of GEF (69 °C) and of the polymer (HPMCAS 121 °C or HPMC 141 °C), as expected. DSC scans of pure GEF and polymer are shown in Figures S6 and S7, respectively.

**Figure 6:**
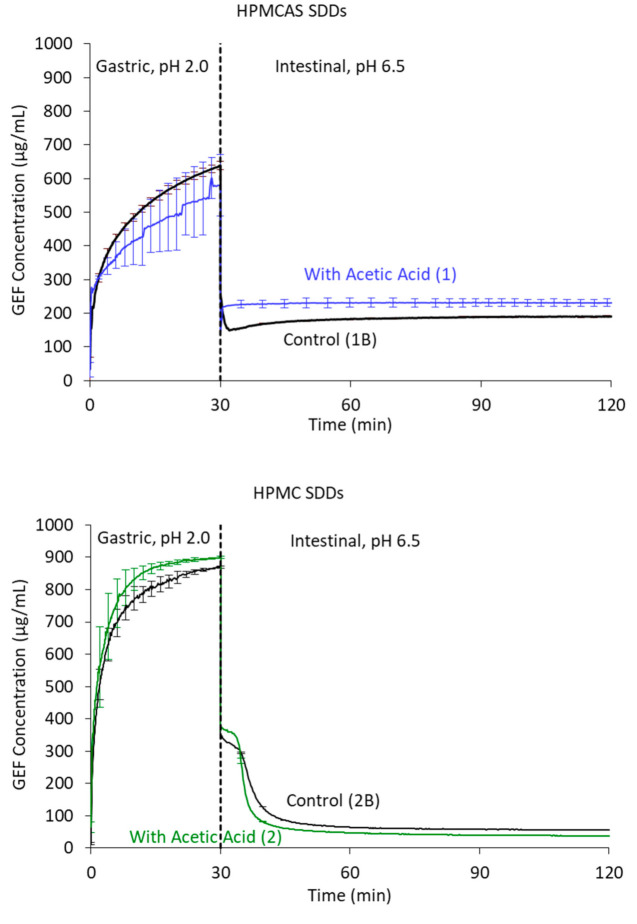
In vitro gastric-to-intestinal transfer dissolution tests for the HPMCAS (**top**) and HPMC (**bottom**) SDDs sprayed from THF:H_2_O (control) and from MeOH:H_2_O with acetic acid as a processing aid.

SDD dissolution performance was analyzed with an in vitro simulated gastric-to-intestinal transfer dissolution test. Such a non-sink transfer test using physiologically relevant pH is often used to rank order different SDDs for formulation selection, or as an input to physiological based pharmacokinetic (PBPK) models to predict absorption. However, in the present study a dissolution test capable of tracking supersaturated drug formulations was used merely to confirm that SDDs made using the acetic acid processing aid in MeOH:H_2_O perform similarly to SDDs of the same composition sprayed from THF:H_2_O. In this test, both dissolution rate (in the simulated gastric portion of the test) and sustainment of supersaturation (in the simulated intestinal portion of the test) were compared. Dissolution profiles for the acetic acid processed vs. control SDDs were similar for each of the two common dispersion polymers—HPMC and for HPMCAS (Figure 6).

## 4. Discussion

### 4.1. Gefitinib Solubility

High energy salt forms of drugs have been used in the pharmaceutical industry to increase the bioavailability by increasing the dissolved drug levels. The same concept is used here to increase solubility in an organic solvent for spray drying. Acetic acid ionizes GEF, which gives it a higher solubility than the GEF-free base in the MeOH:H_2_O blend. MeOH supports the charge transfer due to its high polarity and ability to hydrogen bond with and to solvate the ionic species.

The maximum drug solubility enhancement in spray solvent due to ionization can be estimated by calculating the fraction of ionized drug (DH^+^) in the acid-base equilibrium with a given quantity of acetic acid (Equation (1)). The total solubility is then calculated in Equation (4):

(4) $$S=\frac{S_{0}}{\left(1-F\right)}$$

where *S*_0_ is the intrinsic solubility (i.e., of the un-ionized drug) and *F* is the fraction ionized.

Based on the aqueous p*K*_a_ values of acetic acid (4.75) and of GEF (7.2) [26], two molar equivalents of acetic acid would be expected to result in almost complete protonation of GEF, leading to a very large potential enhancement (200–300× over the free base solubility in water—a solubility enhancement that would likely be limited by the solubility product (*K*_sp_) of the GEF acetate salt. However, it has been reported that carboxylic acids are much weaker in MeOH (~5 orders of magnitude) than in water, while tertiary amine p*K*_a_*s*, such as that on GEF, change very little in MeOH vs. water [29]. Therefore, in MeOH and MeOH-rich solvents, acetic acid might be expected to have a much higher p*K*_a_ than in water and be less effective at protonating the amine group of GEF. Indeed, NMR measurements of the fraction GEF ionized in the presence of several molar equivalents of acetic acid suggest a ∆ p*K*_a_ (p*K*_a,d_ − p*K*_a,a_) of about −0.9 in pure MeOH, and about 0.1 in 80:20 MeOH:H_2_O solutions, versus a ∆ p*K*_a_ of about 2.5 in water. Here the addition of some water to the MeOH is useful, as the intrinsic drug solubility in MeOH is not much decreased with the addition of 10–20% water, and the addition of water increases the strength of acetic acid in MeOH by about an order of magnitude, increasing the fraction drug ionized and therefore the total solubilized drug concentration.

GEF solubility data (Figure 2) shows that in 80:20 MeOH:H_2_O, approximately 20 mg/mL of acetic acid can be used to increase the solubility of GEF about 10-fold, from just under 5 mg/mL to 50 mg/mL. The NMR data suggests that most of this increase is due to ionization of the drug. That is, there is an approximately 5-fold enhancement (cf. Equation (3)) due to 80% protonation of GEF. There is an additional smaller enhancement in solubility (~2-fold) due to acetic acid changing the solvent character of MeOH:H_2_O to increase either the GEF intrinsic (free base) solubility ([D]_s_ in Equation (3)), and/or slightly changing the ∆ *pK*_a_ relative to what it is in MeOH:H_2_O without acetic acid.

All solubility experiments were performed with excess solids of the MeOH hemisolvate (Form II), as confirmed by PXRD of solids isolated at the end of the experiments (Figure S2). Form II is less soluble in methanolic solutions than the Form I starting material. Additional peaks were observed for some samples (Figure S2) containing acetic acid, which likely result from the precipitation of salts from the wet solids during sample preparation and analysis.

The GEF concentration in the spray solution used to make SDDs (18 mg/mL in 80:20 MeOH:H_2_O with 4.9 mg/mL acetic acid) was greater than the measured equilibrium solubility of 14.8 mg/mL (Figure 2). This is the result of the higher solubility of Form I in methanolic solutions and maintenance of supersaturation (relative to the more stable Form II) over the course of spray drying. This supersaturation is likely enabled by the relative low supersaturation (concentration/solubility = 1.2) and possibly aided by the presence of polymer, which may inhibit precipitation and/or increase solubility.

Methanol and its blends with water were chosen as the solvents for the present work due to their ability to solubilize ionized drug species. In addition, MeOH has a low boiling point (65 °C) and heat of vaporization (37.6 kJ/mol) to facilitate solvent removal, and also dissolves the most common polymers used for SDDs, including HPMCAS, Eudragit L, PVP VA64, PVP K30, and, with the addition of water, HPMC. These advantages, along with relatively low cost, make MeOH a good choice for the present application.

Acetic acid was selected as the acidic processing aid because of its high volatility and ability to form high energy salts with weakly basic APIs that have low solubility in MeOH. After the solutions are spray dried, the acetic acid is rapidly removed during secondary drying. It is a low-toxicity class III solvent with a high ICH limit (0.5 wt%) for pharmaceutical products [28]. Note that a stronger acid can be chosen in order to achieve a higher drug protonation. For example, formic acid has a p*K*_a_ about one unit lower than acetic acid, and might be useful for weakly basic drugs having a lower p*K*_a_ than GEF.

### 4.2. SDD Characterization and Performance

SDD morphology (by SEM), the amorphous state of the API (by PXRD), and homogeneity (by DSC) were all similar for SDDs made using acetic acid-containing spray solutions relative to the control SDDs made from THF:H_2_O. In vitro dissolution performance comparing the acetic acid processed SDDs to the control SDDs showed no appreciable differences in dissolution rate or extent for either HPMC and HPMCAS SDDs. As for many drugs, HPMCAS SDDs of GEF show superior supersaturation sustainment relative to other polymers, including HPMC [30,31]. The in vitro characterization demonstrates that the acetic acid process appears to not significantly impact GEF SDD characteristics, and our expectation would be that SDDs made with the control vs. acetic-acid processes would provide similar performance in vivo.

### 4.3. Throughput Increase and Material Savings

Solubility enhancement of weakly basic APIs through the aid of acetic acid allows for significantly improved manufacturing throughput without having to resort to less desirable solvents, saving time, material, and cost. The overall throughput from spray drying is the product of dissolved solids concentration and flow rate into the spray dryer. The main limitation for throughput is often the solubility of the drug in the solvent. High solution viscosity due to high polymer concentrations can limit throughput in some applications, though this is not common for APIs having poor organic solubility. With relatively small amounts of added acetic acid, the viscosity is minimally impacted, allowing for similar spray drying conditions used in the absence of acetic acid, but with the benefit of increased throughput. The viscosity measurements and limits for each scale of spray drying, shown in Figures S9 and S10, confirm that the viscosity is minimally impacted using small amounts of acetic acid.

A maximum throughput calculation using GEF as an example (Table 3) suggests that using acetic acid as a processing aid can dramatically decrease the process time and solvent usage due to increased API solubility in the solvent. Using acetic acid as a processing aid, solvent consumption can be decreased by 3.8× and 10.9× in MeOH and 80:20 MeOH:H_2_O, respectively. Spray drying time is decreased 3.6-fold and 9-fold in MeOH and 80:20 MeOH:H_2_O, respectively, using acetic acid as the processing aid. For polymers that impart high viscosity, such as HPMCAS and HPMC, one might start to encounter viscosity limitations for pressure nozzles at 9–12 wt% polymer due to incomplete atomization. For polymers with lower solution viscosities, such as PVPVA, viscosity would be less likely to limit throughput. The decrease in solvent consumption and time allows for significant savings in time and cost. The use of smaller amounts of less harmful solvents likewise reduces the environmental impact of processing. This is garnering more attention in the pharmaceutical industry as it works towards sustainability.

Acetic acid has the potential to be a valuable processing aid for a significant number of drug molecules requiring bioavailability enhancement. In particular, an analysis of a historical Lonza portfolio of 1318 APIs formulated for bioavailability suggest that 60% of these are weak bases (i.e., at least one basic p*K*_a_ > 2). Although the approach was demonstrated here using a single model drug, it is expected to apply to other basic APIs, especially those with higher p*K*_a_ values that will be largely protonated by acetic acid. This technology can be applied for a range of dispersion excipients including both ionizable and non-ionizable polymers.

Note that an approach analogous to the one described here can be applied to weakly acidic APIs. Specifically, basic processing aids, such as ammonia in methanol or ammonium hydroxide, can be used to deprotonate acidic APIs to improve solubility in methanolic solutions similarly to what was done here to ionize a basic drug [32].

## 5. Conclusions

Using acetic acid in small quantities as a processing aid for spray drying from MeOH and MeOH:H_2_O blends can significantly increase solubility for weakly basic APIs. In this study we demonstrated the technology with the model compound gefitinib (GEF), and successfully manufactured amorphous SDDs using two different dispersion polymers and typical processing conditions. GEF solubility was increased 4-fold and 10-fold in MeOH and MeOH:H_2_O, respectively, using small amounts of acetic acid. The increase is due primarily to protonation of GEF, i.e., acetate salt formation. Acetic acid is volatile and easily removed to below ICH limits to regenerate the starting GEF free base. The SDDs manufactured with acetic acid in MeOH:H_2_O were comparable to control formulations manufactured from THF, having similar morphology, glass transition temperature, crystallinity, and dissolution performance. Acetic acid in methanolic solutions is a safer and more environmentally sound option than some less desirable solvents, including chlorinated solvents, for achieving commercially relevant throughputs resulting in equivalent product quality. With the increasing number of brick dust compounds, this technology can be enabling for manufacturing spray dried dispersions of weakly basic APIs to achieve bioavailability enhancement.

## 6. Patents

Patent pending related to the work reported in this manuscript titled: Acetic Acid as a Processing Aid in Spray Drying for Basic Drugs.

## Figures and Tables

**Table 1:** Summary of the spray conditions for the 25:75 (*w*/*w*) GEF:polymer formulations. Lots 1–2 are the formulations manufactured using acetic acid and lots 1B-2B are the controls made from THF: H_2_O.

Lot ID	Dispersion Polymer	Solvent Composition		Dissolved Solids (Drug + Polymer) Concentration (wt%)	GEF Concentration	
		Solvent	(wt%)		(wt%)	(mg/mL)
1	HPMCAS	MeOH	84.5	8.7	2.17	19.6
		H_2_O	14.8			
		acetic acid	0.7 ^a^			
2	HPMC	MeOH	79.5	8.6	2.15	19.5
		H_2_O	19.9			
		acetic acid	0.6 ^b^			
1B	HPMCAS	THF	95	8.7	2.18	21.3
		H_2_O	5			
2B	HPMC	THF	80	8.6	2.15	21.3
		H_2_O	20			

^a^ 2.1 molar equivalents relative to GEF and 5.6 mg/mL of acetic acid in the solvent blend. ^b^ 1.9 molar equivalents relative to GEF and 5.1 mg/mL of acetic acid in the solvent blend.

**Table 2:** Fraction ionized and calculated Δ p*K*_a_ for two acetic acid concentrations in methanol and 80:20 methanol:H_2_O.

Acetic Acid Conc. (mg/mL)	MeOH		80:20 MeOH:H_2_O	
	Fraction Drug Ionized	Calc. Δ p*K*_a_	Fraction Drug Ionized	Calc. Δ p*K*_a_
13.8	0.60	−0.89	0.78	0.05
21.8	0.68	−0.74	0.81	0.13

**Table 3:** Throughput estimate for 100 kg of 25% GEF SDD assuming maximum API solubility of GEF shown in Figure 2, assuming a solution feed rate of 50 kg/h.

Parameter		MeOH		80:20 MeOH:H_2_O	
		No Acetic Acid	+Acetic Acid ^a^	No Acetic Acid	+Acetic Acid ^a^
GEF Solubility	(mg/mL)	5	19.3	4.6	50
	(wt%)	0.62	2.2	0.54	4.8
Dissolved Solids (wt%)		2.5	8.9	2.2	19.4
Solvent Volume (L)		4970	1300	5430	500
Spray Time (Hours)		80.6	22.5	92.6	10.3

^a^ 21.8 mg/mL acetic acid.

## Data Availability

Microsoft Excel worksheets of the data in this study are available from the corresponding author upon request.

## Supplementary Materials

The following are available online at https://www.mdpi.com/1999-4923/14/3/555/s1, Supplementary Material S1: GC Method Details, Figure S1: PXRD of as-received GEF from LC Labs, lot BGF-107 (top, red), and recrystallized from MeOH, IRD-631-89 (bottom, purple), Figure S2: PXRD of solids recovered from saturated solutions of gefitinib in MeOH, 9:1 MeOH:H_2_O, and 8:2 MeOH:H_2_O with varying amounts of acetic acid. Areas denote (*) show differences with addition of 200 μL acetic acid while areas marked with (^) show differences with additional water, Figure S3: Chemical shift versus acetic acid concentration in the titration of Gefitinib with acetic acid-d3 in methanol-d4, Figure S4: Drying curve of methanol and acetic acid for both tray drying conditions for the formulation 25/75 GEF/HPMCAS (lot 1) sprayed using the acetic acid processing aid in MeOH:H_2_O, Figure S5: Drying curve of methanol and acetic acid for both tray drying conditions for the formulation 25/75 GEF/HPMC (lot 2) sprayed using the acetic acid processing aid in MeOH:H_2_O, Figure S6: Representative heat flow thermogram of GEF after melt-quench from the molten state, Figure S7: Reversing heat flow showing Tg event for HPMCAS and HPMC E3, Figure S8: DSC data for the 4 SDDs, showing the reversing non-reversing and total heat flow traces, Figure S9: Viscosity data for HPMCAS in different solvents, and the estimated viscosity limits for sufficient atomization for different scales of spray drying, Figure S10: Viscosity data for HPMC in different solvents and the estimated viscosity limits for sufficient atomization for different scales of spray drying, Table S1: Patents associated with each form of GEF, Table S2: Samples of GEF prepared by various methods, and their forms.
