# Correction: Adam et al. Acetic Acid as Processing Aid Dramatically Improves Organic Solvent Solubility of Weakly Basic Drugs for Spray Dried Dispersion Manufacture. *Pharmaceutics* 2022, *14*, 555

**DOI:** 10.3390/pharmaceutics14061225

**Published:** 2022-06-09

**Authors:** Molly S. Adam, Warren K. Miller, Amanda M. Pluntze, Aaron M. Stewart, Jonathan L. Cape, Michael E. Grass, Michael M. Morgen

**Affiliations:** Global Research & Development, Lonza, Bend, OR 97703, USA; molly.adam@lonza.com (M.S.A.); warren.miller@lonza.com (W.K.M.); amanda.pluntze@lonza.com (A.M.P.); aaron.stewart@sagerx.com (A.M.S.); jon.cape@lonza.com (J.L.C.); michael.grass@lonza.com (M.E.G.)

**Figure Legend**

In the original publication [1], there was a mistake in the legend for Figure 2. The label for the blue line was labeled as “80:20 MeO:H_2_O”: this methanol chemical formula was incorrect. This is corrected to “80:20 MeOH:H_2_O”, as appears below.

**Error in Table**

In the original publication, there was a mistake in Table 3, as published. The values of the throughput estimates for the gefitinib weight percent solubility, dissolved solids, solvent volume, and spray time were incorrect. The label for the rightmost column also had the incorrect methanol chemical formula, as published. The corrected Table 3 appears below.

**Text Correction**

There was an error in the original publication. In the text of the Throughput Increase and Material Savings section, values for the solvent consumption decrease and spray drying time decrease were incorrect.

A correction has been made to paragraph number two of the Throughput Increase and Material Savings sub-section of the Discussion section. 

A maximum throughput calculation using GEF as an example (Table 3) suggests that using acetic acid as a processing aid can dramatically decrease the process time and solvent usage due to increased API solubility in the solvent. Using acetic acid as a processing aid, solvent consumption can be decreased by 3.8× and 10.9× in MeOH and 80:20 MeOH:H_2_O, respectively. Spray drying time is decreased 3.6-fold and 9-fold in MeOH and 80:20 MeOH:H_2_O, respectively, using acetic acid as the processing aid. For polymers that impart high viscosity, such as HPMCAS and HPMC, one might start to encounter viscosity limitations for pressure nozzles at 9–12 wt% polymer due to incomplete atomization. For polymers with lower solution viscosities, such as PVPVA, viscosity would be less likely to limit throughput. The decrease in solvent consumption and time allows for significant savings in time and cost. The use of smaller amounts of less harmful solvents likewise reduces the environmental impact of processing. This is garnering more attention in the pharmaceutical industry as it works towards sustainability.

The authors apologize for any inconvenience caused and state that the scientific conclusions are unaffected. This correction was approved by the Academic Editor. The original publication has also been updated.

## Figures and Tables

**Figure 2:**
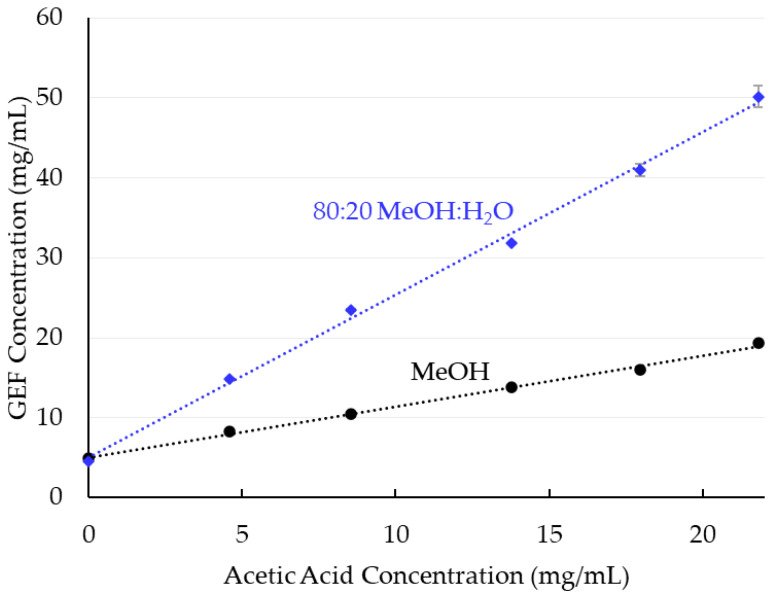
Solubility of GEF in MeOH and 80:20 MeOH:H_2_O as a function of acetic acid concentration.

**Table 3:** Throughput estimate for 100 kg of 25% GEF SDD assuming maximum API solubility of GEF shown in Figure 2, assuming a solution feed rate of 50 kg/h.

Parameter		MeOH		80:20 MeOH:H_2_O	
		No Acetic Acid	+Acetic Acid ^a^	No Acetic Acid	+Acetic Acid ^a^
GEF Solubility	(mg/mL)	5	19.3	4.6	50
	(wt%)	0.62	2.2	0.54	4.8
Dissolved Solids (wt%)		2.5	8.9	2.2	19.4
Solvent Volume (L)		4970	1300	5430	500
Spray Time (Hours)		80.6	22.5	92.6	10.3

^a^ 21.8 mg/mL acetic acid.
