# Supplementary material for: Acetic Acid as Processing Aid Dramatically Improves Organic Solvent Solubility of Weakly Basic Drugs for Spray Dried Dispersion Manufacture
Source: Pharmaceutics. 2022 Mar 2;14(3):555. doi: 10.3390/pharmaceutics14030555 (PMC8950584; doi:10.3390/pharmaceutics14030555)
Supplement: Supplementary file 1 [file pharmaceutics-14-00555-s001.zip › pharmaceutics-1601005-supplementary.pdf]

pharmaceutics

Supplementary Materials: Acetic Acid as Processing Aid Dramatically Improves Organic Solvent Solubility of Weakly Basic Drugs for Spray Dried Dispersion Manufacture

Molly S. Adam, Warren K. Miller, Amanda M. Pluntze, Aaron M. Stewart, Jonathan L. Cape, Michael E. Grass and Michael M. Morgen

Supplementary Material S1: GC Method Details

Acetic acid and MeOH

A known amount of sample (ca. 200–300 mg) was placed in a 20mL headspace vial and dissolved in 4mL of dimethylacetamide. Samples were heated with shaking for 10 min at 118 °C, pressurized to 20 psi for 0.33 min with nitrogen, extracted into the 1 mL sample loop at 120 °C and 125 °C transfer line, and followed by injection onto the DB-624 UI column (20 m × 0.18 mm ID × 1.0 μm film thickness) through a 2 mm direct deactivated inlet liner set at 180 °C. Chromatography was performed in constant flow mode at 1.6 mL/min with hydrogen as the carrier gas, and analyzed with a flame ionization detector at 260 °C using nitrogen as the make-up gas. The oven cycle began with a 30 °C/min ramp from 40 to 70 °C, followed by a 5 °C/min ramp to 100 °C and then 30 °C/min to 220 °C with a 0.5 min isothermal hold for a total runtime of 14.5 min.

THF

A known amount of sample (ca. 40–100 mg) was placed in a 20 mL headspace vial and dissolved in 4 mL of dimethylacetamide. Samples were heated for 30 min at 105 °C, pressurized to 20 psi for 0.33 min with nitrogen, and extracted into the 1 mL sample loop at 110 °C followed by injection onto the DB-624 column (30 m × 0.32 mm ID x 1.8 μm film thickness) with a 1 min injection time into a 2 mm direct deactivated inlet liner set at 180 °C. Chromatography was performed in constant flow mode at 5 mL/min with hydrogen as the carrier gas, and analyzed with a flame ionization detector at 260 °C. The oven cycle began at 40 °C for 2 min, followed by a 50 °C/min ramp to 225 °C with a 0.5 min isothermal hold for a total runtime of 6.2 min.

Supplementary Material S2: PXRD

Table S1. Patents associated with each form of GEF.

Form	Type	Described in
Form 1	Anhydrous	EP 1480650B1
Form 2	MeOH hemisolvate	EP 1480650B1
Form 3	DMSO monosolvate	EP 1480650B1
Form 5	Trihydrate	EP 1480650B1
Form 6	Monohydrate	WO2006090413A1

Table S2. Samples of GEF prepared by various methods, and their forms.

Sample	Lot/Sample	Form
LC Labs API	BGF-106 and BGF-107	Form 1 + Peaks 6.5, 8.2, 13.0, 16.6, 20.2 (possibly consistent with Form 2)
Precipitated from MeOH	IRD631-89	Form 2

Solids saturated in MeOH + 200 mL acetic acid	S6	Form 2 + Peaks 5 and 15.1
Solids saturated in 8:2 MeOH:water with some acetic acid	S15, s16	Form 2 w/ several changed peaks

The crystalline gefitinib used for this work was from LC Labs. Both lots (BGF-106 and BGF-107) appear to consist of mostly anhydrous Form 1 with some additional peaks that may be consistent with the presence of MeOH hemisolvate Form 2 (Figure S6).

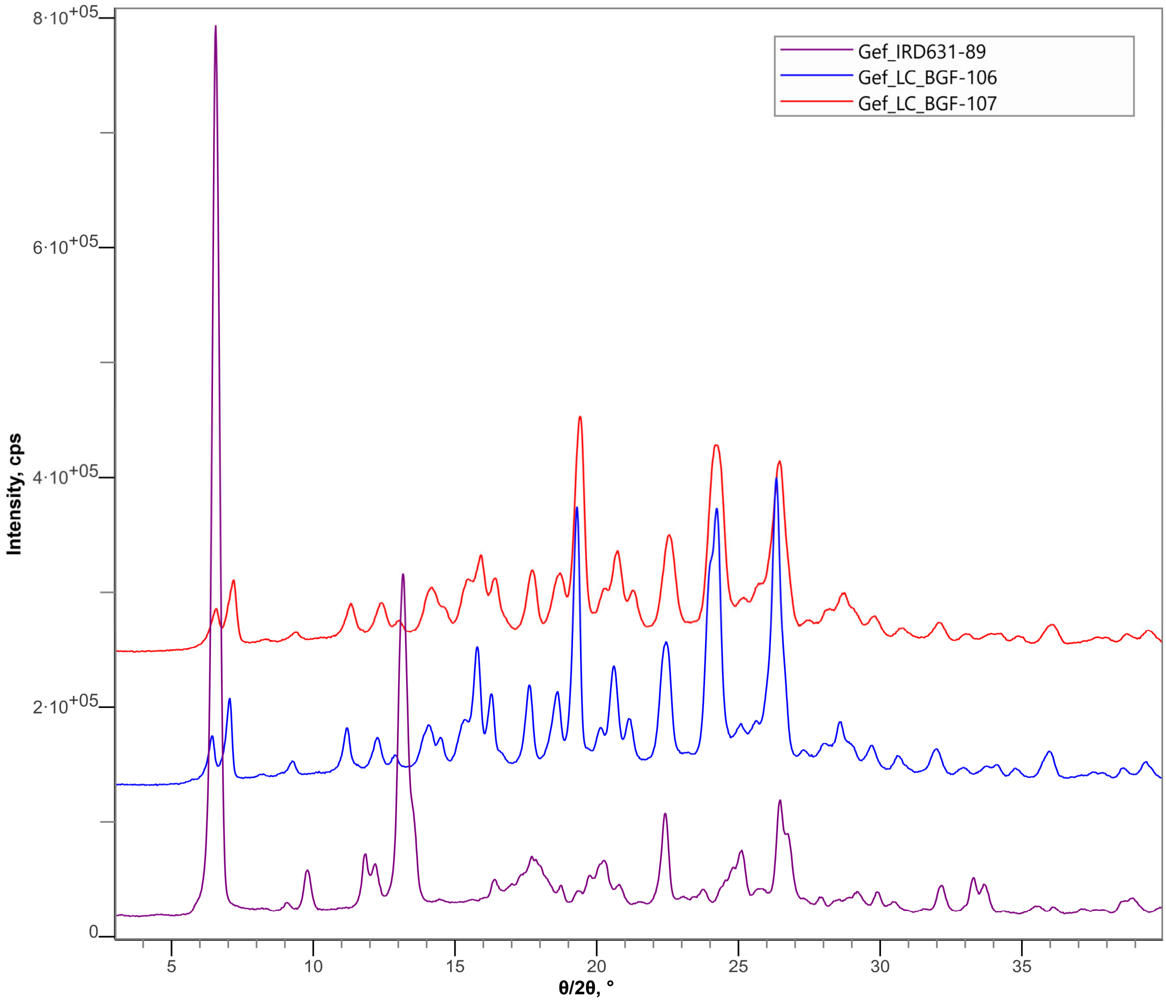

Figure S1. XRD of as-received GEF from LC labs, lot BGF-106 (middle, blue), BGF-107 (top, red), and recrystallized from MeOH, IRD-631-89 (bottom, purple).

Gefitinib (4.25 g) was dissolved in 200 mL refluxing MeOH. After cooling to room temperature and standing for two days, crystals of gefitinib were collected and dried by suction. Additional crystals were obtained by reducing the volume of methanol to 100mL by roto-evaporation, chilling to −20°C and adding a few seed crystals. Crystallization occurred within minutes and crystals were collected by filtration. The total yield was 3.14 g, 74%. This sample recrystallized from MeOH is consistent with the MeOH hemisolvate Form 2 as expected.

In experiments with saturated GEF in MeOH or MeOH/water blends with acetic acid, the resulting solids mostly match with Form 2, but with key changes to the diffractograms. With the addition of at least 200 μL acetic acid, peaks appear at 5.0 and 15.1° 2theta. In samples with at least some acetic acid and 20% water, several peaks appear or shift (Figure S7). These peaks do not correlate to any of the described forms of GEF.

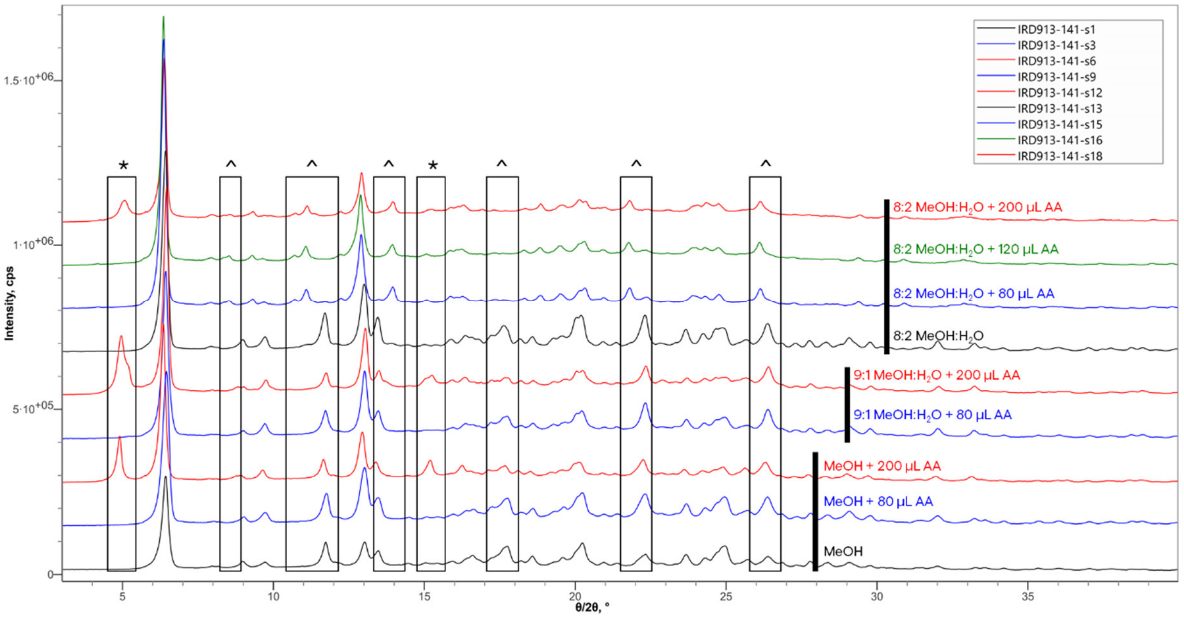

Figure S2. PXRD of solids recovered from saturated solutions of gefitinib in MeOH, 9:1 MeOH:H_2O, and 8:2 MeOH:H_2O with varying amounts of acetic acid. Areas denoted (*) show differences with addition of 200μL acetic acid while areas marked with (^) show differences with additional water.

Supplementary Material S3: NMR Titration

Titrations performed in deuterated solvents were used to map the chemical shift (δ) versus acetic acid concentration profiles that characterize the shift in characteristic resonances with protonation. For this study the δ = 2.12 ppm resonance of the morpholine moiety was used as an indicator of protonation. Dashed lines in these figures are polynomial fits to the data that were used to establish endpoint chemical shift values at 100% protonation. Estimation of the degree of protonation in experimental samples from protic solvents were based upon these titrations, utilizing endpoint chemical shifts to represent 0 and 100% protonation and assuming a linear variation in chemical shift with the degree of protonation between endpoint values.

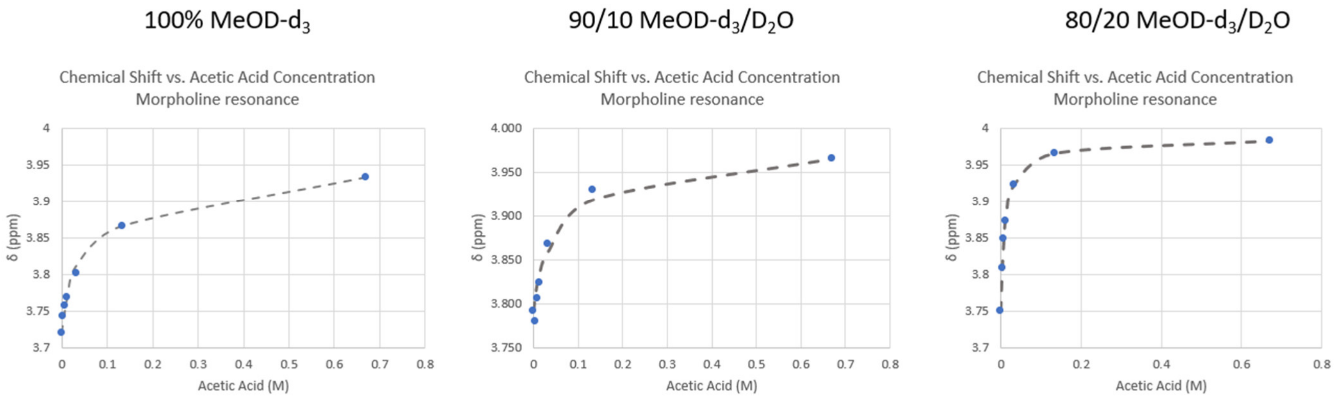

Figure S3. Chemical shift versus acetic acid concentration in the titration of GEF with acetic acid-d_3 in methanol-d_4.

Supplementary Material S4: Secondary Drying Results

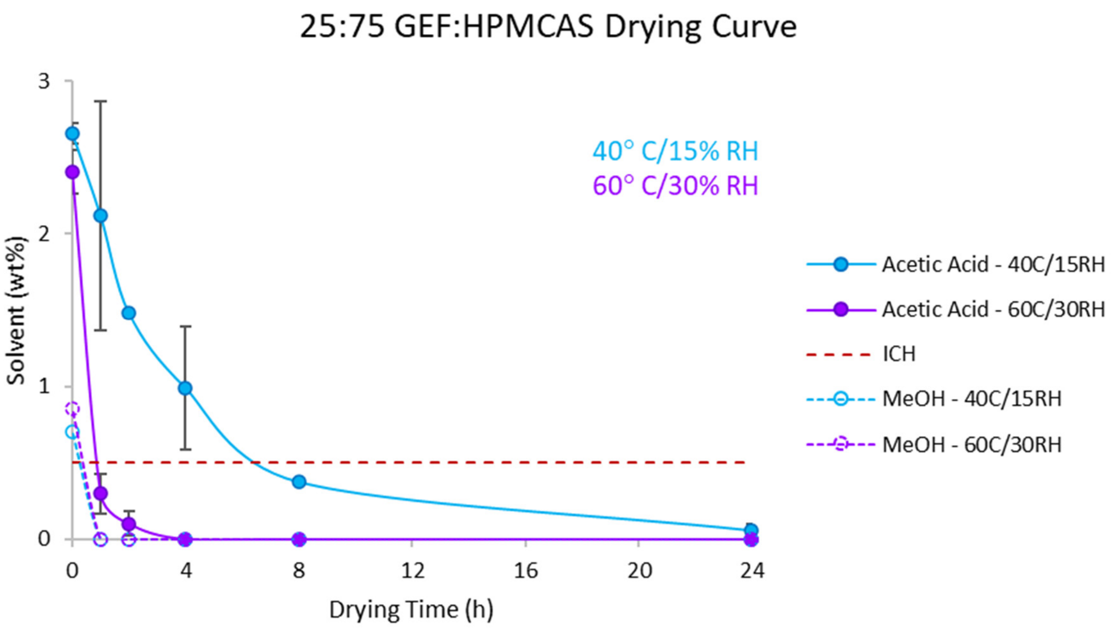

Figure S4. Drying curve of methanol and acetic acid for both tray drying conditions for the formulation 25:75 GEF:HPMCAS (lot 1) sprayed using the acetic acid processing aid in $MeOH:H_2O$.

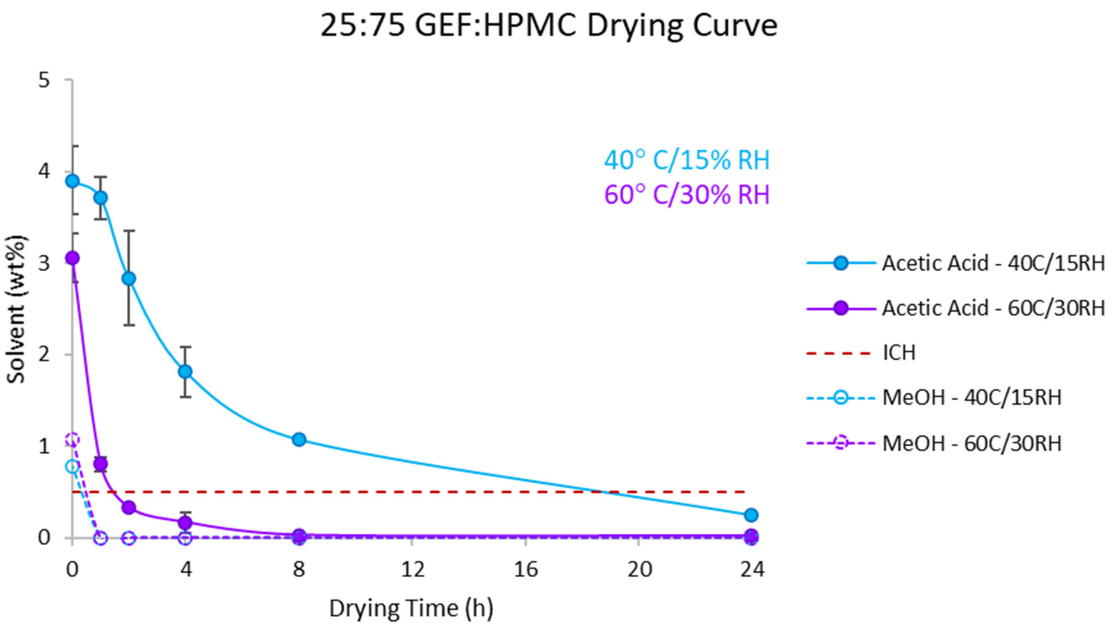

Figure S5. Drying curve of methanol and acetic acid for both tray drying conditions for the formulation 25:75 GEF:HPMC (lot 2) sprayed using the acetic acid processing aid in $MeOH:H_2O$.

Supplementary Material S5: DSC

To determine the T_g of GEF, a DSC sample was heated at 50 °C/min until the melt event was completed. The pan was removed from the DSC furnace while in its molten state, and put into liquid nitrogen to rapidly quench it in the glassy state. The sample was then scanned from 0–220 °C at 2.5 °C/min with ± 1.5 °C/min modulation. The total heat

flow in **Error! Reference source not found.** shows the T_g of 69 °C, recrystallization at 115 °C, and T_m of 192 °C.

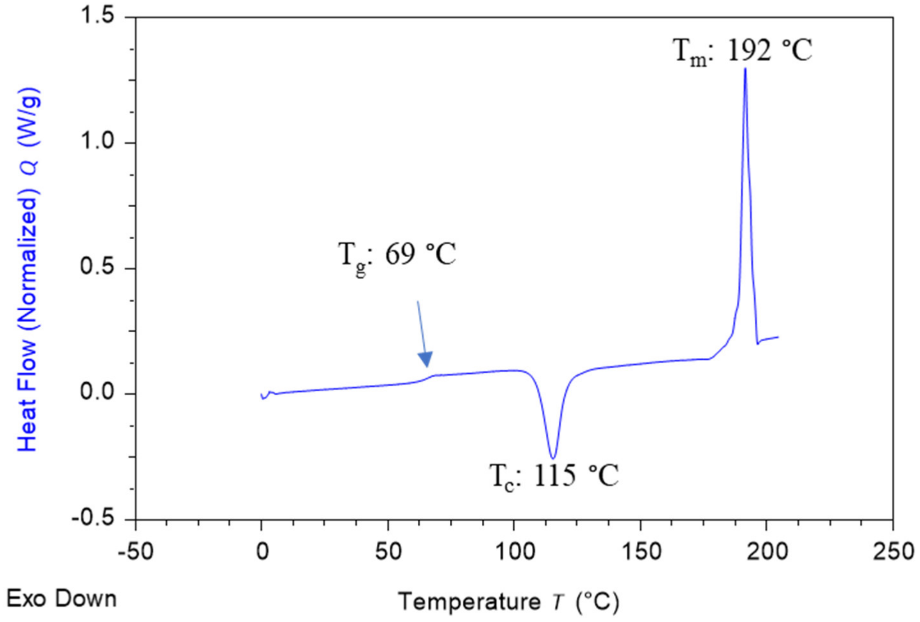

Figure S6. Representative heat flow thermogram of GEF after melt-quench from the molten state.

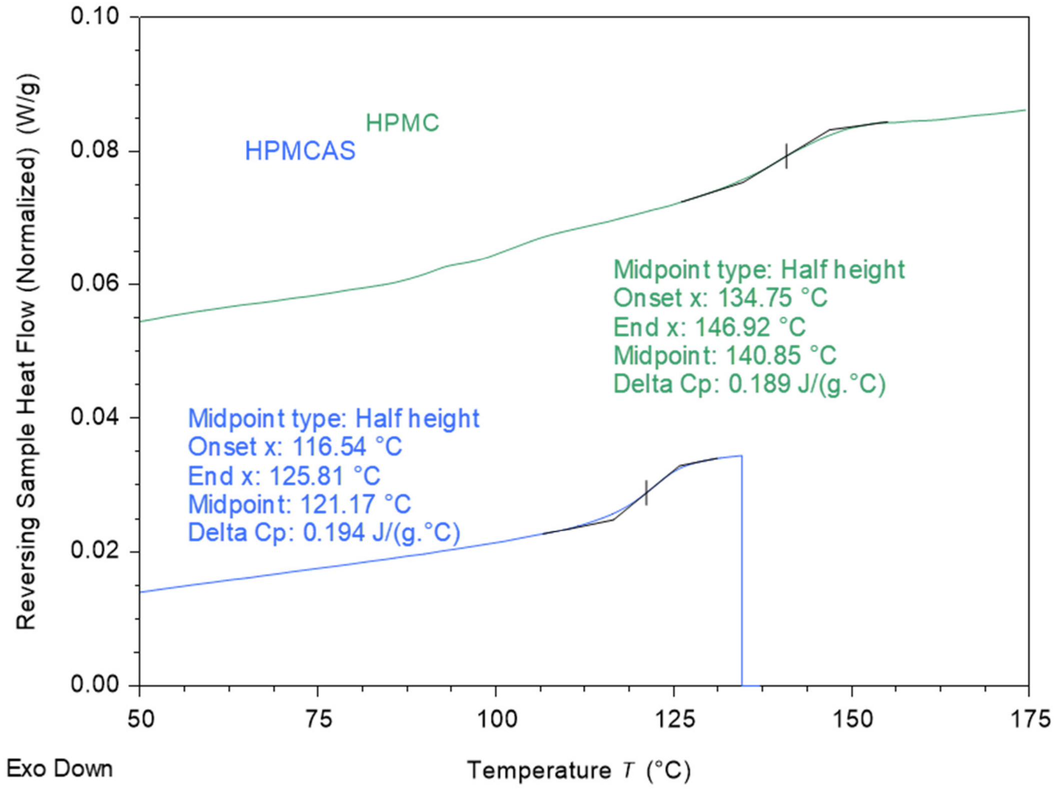

Figure S7. Reversing heat flow showing T_g event for HPMCAS and HPMC.

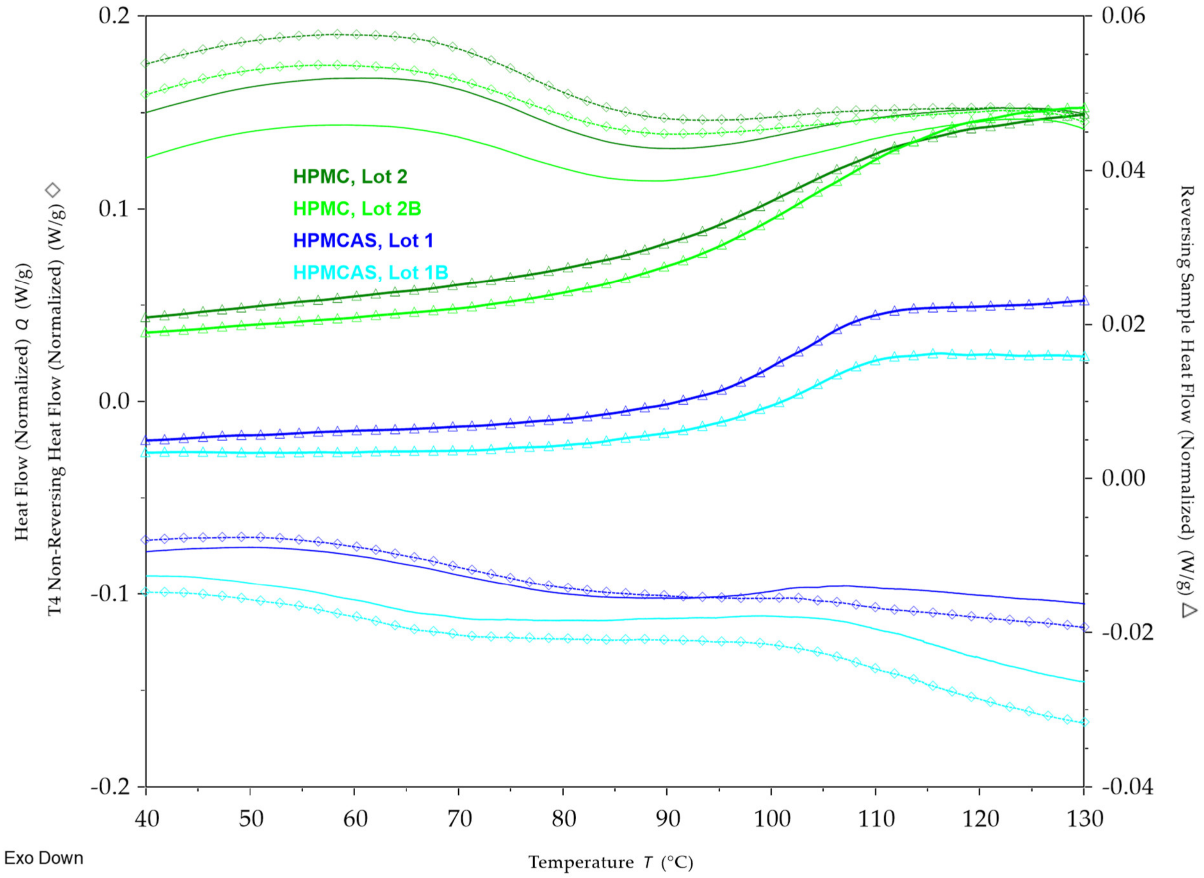

Figure S8. DSC data for the 4 SDDs, showing the reversing (triangles), non-reversing (diamonds), and total (solid line) heat flow traces. Traces are offset for clarity.

Supplementary Material S6: Viscosity Measurements

Viscosity measurements are shown below for each polymer in methanol, methanol:acetic acid blends, and pure acetic acid (water was included in the solvent blends for HPMC in order to dissolve the polymer). The horizontal dotted lines show rough viscosity limits for robust atomization based on historical data at Lonza-Bend. Adding 10% *w*/*w* acetic acid does not significantly increase viscosity of HPMCAS in methanol or of HPMC in MeOH:H_2O. Pure acetic acid has a much higher viscosity that would limit polymer concentration and throughput especially at commercial scale. In the present approach, the small amount of acetic acid in methanol or MeOH:H_2O (<<10%wt./wt.) allows for the increased throughput without the viscosity limitation.

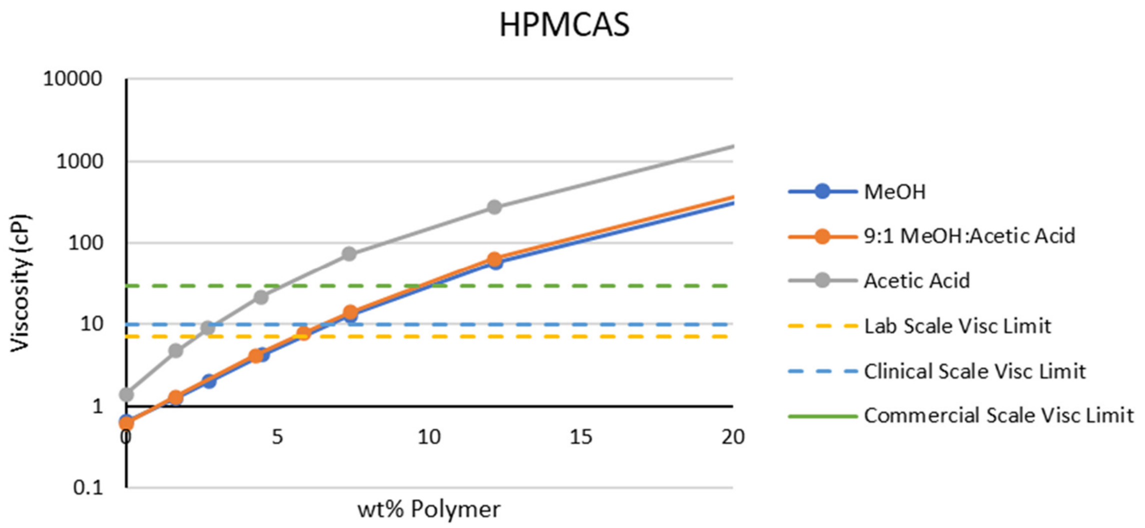

Figure S9. Viscosity data for the polymer HPMCAS in different solvents and the estimated viscosity limits for sufficient atomization for different scales of spray drying. All solvent blend ratios are wt./wt.

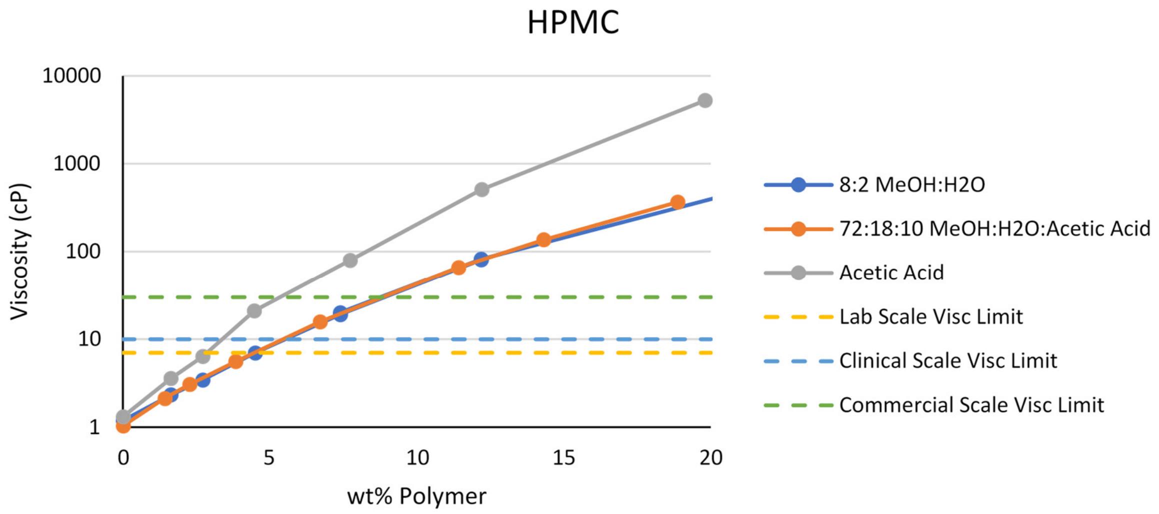

Figure S10. Viscosity data for the polymer HPMC in different solvents and the estimated viscosity limits for sufficient atomization for different scales of spray drying. All solvent blend ratios are wt./wt.
